# Novel space alters theta and gamma synchrony across the longitudinal axis of the hippocampus

**DOI:** 10.3389/fnsys.2013.00020

**Published:** 2013-06-25

**Authors:** Stephanie C. Penley, James R. Hinman, Lauren L. Long, Etan J. Markus, Monty A. Escabí, James J. Chrobak

**Affiliations:** ^1^Department of Psychology, University of ConnecticutStorrs, CT, USA; ^2^Department of Biomedical Engineering, University of ConnecticutStorrs, CT, USA; ^3^Department of Electrical and Computer Engineering, University of ConnecticutStorrs, CT, USA

**Keywords:** memory, encoding, oscillations, coherence, rodent

## Abstract

Hippocampal theta (6–10 Hz) and gamma (25–50 Hz and 65–100 Hz) local field potentials (LFPs) reflect the dynamic synchronization evoked by inputs impinging upon hippocampal neurons. Novel experience is known to engage hippocampal physiology and promote successful encoding. Does novelty synchronize or desynchronize theta and/or gamma frequency inputs across the septotemporal (long) axis of the hippocampus (HPC)? The present study tested the hypothesis that a novel spatial environment would alter theta power and coherence across the long axis. We compared theta and gamma LFP signals at individual (power) and millimeter distant electrode pairs (coherence) within the dentate gyrus (DG) and CA1 region while rats navigated a runway (1) in a familiar environment, (2) with a modified path in the same environment and (3) in a novel space. Locomotion in novel space was related to increases in theta and gamma power at most CA1 and DG sites. The increase in theta and gamma power was concurrent with an increase in theta and gamma coherence across the long axis of CA1; however, there was a significant decrease in theta coherence across the long axis of the DG. These findings illustrate significant shifts in the synchrony of entorhinal, CA3 and/or neuromodulatory afferents conveying novel spatial information to the dendritic fields of CA1 and DG targets across the long axis of the HPC. This shift suggests that the entire theta/gamma-related input to the CA1 network, and likely output, receives and conveys a more coherent message in response to novel sensory experience. Such may contribute to the successful encoding of novel sensory experience.

## Introduction

Neuroimaging data highlight the differential activation of the hippocampus (HPC) along its anterior-posterior axis (Stern et al., 1996; Strange et al., 1999; Binder et al., 2005; Ta et al., 2011 for review). The posterior pole of the HPC in primates is analogous to the septal pole in rodents, and correspondingly the anterior to the temporal pole. These neuroimaging studies complement a history of rodent studies supporting functional segregation across the septotemporal (long) axis of the HPC (Hughes, 1965; Sinnamon et al., 1978; Moser and Moser, 1998; Bannerman et al., 2004).

Functional segregation along the long axis of the HPC likely originates in the entorhinal cortex (EC). Topographically organized entorhinal inputs define broad septotemporal domains within the HPC (Ruth et al., 1982; Dolorfo and Amaral, 1998a,b; Witter, 2007) conveying clusters of neocortical associative input to different longitudinal levels (Suzuki and Amaral, 1994; Burwell and Amaral, 1998; Lavenex and Amaral, 2000). In contrast, intrinsic connections (e.g., CA3 to CA1, hilar mossy cells to granule cells) diverge extensively and may support functional integration (Lavenex and Amaral, 2000; Kondo et al., 2008).

Novel sensory experience engages the HPC (Wilson and McNaughton, 1993; Knight, 1996; Stern et al., 1996; Law et al., 2005). Studies by Vinogradova and colleagues (Vinogradova, 2001 for review) illustrated the response to novelty and habituation of network neurons to repeated presentation of sensory cues. Nitz and McNaughton (2004) observed differential modulation of pyramidal cells and interneurons in CA1 and DG in rodents navigating in novel vs. familiar environments. Few studies have described the dynamics of theta, or variation across the long axis, in response to novel experience. Importantly, task contingencies (Kentros et al., 2004) as well as simply locomotor speed modify the discharge rate of hippocampal neurons, as well as the amplitude and frequency of theta and gamma local field potentials (LFPs) (Nitz and McNaughton, 2004; Chen et al., 2011; Hinman et al., 2011). Thus, subtle changes in behavior in response to novelty can confound the linkage of sensory or associative processes to physiological events (Buzsáki et al., 1979).

**Figure 1:**
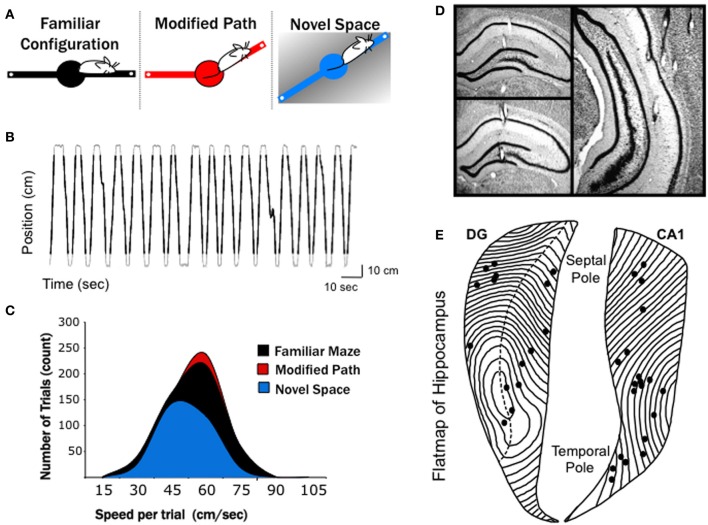
**Experimental conditions and electrode positions. (A)** A series of three, 10-min recordings were obtained from each rat over a 4 days period in: (1) in familiar space, (2) along a modified path in familiar space, and (3) in novel space. The black (familiar), red (modified), and blue (novel) color codes are maintained in all figures to differentiate conditions. The familiar maze configuration consisted of a finely textured linear track placed in a generally white room. On the following day the rats where placed on a modified path in the same room, with one arm altered 30°. Two days later (following 1 day of retraining on the familiar configuration) the rats were run on a coarsely textured linear track in a novel space. **(B)** The position of a rat along the 140-cm linear track over time during 33 consecutive trials is shown. Black lines overlaid on the gray trace indicate the portion of each track traversal that was considered as an individual trial. A threshold 14 cm from either end of the linear track was used to eliminated data at the ends of the runway (see Methods). **(C)** Distribution of mean trial speeds for each recording condition across all animals, with the average speed of trial in the novel space significantly slower than both the familiar maze and modified path conditions. Note fewer total running trials were used for the novel condition due to slower running speeds and longer pauses at the end of each arm during the 10-min recording (see Results). **(D)** Photomicrographs of representative recording sites in septal dentate gyrus, septal CA1, and mid-septotemporal CA1. **(E)** Flatmap representation of the hippocampal formation with all recording locations indicated by dots. The septal pole of the hippocampus is located at top and the temporal pole at bottom.

Our laboratory is focused on septotemporal variation in theta and gamma and asks the question, what, if any, conditions synchronize the theta signal across the long axis? Alterations in theta LFPs largely reflect variation in synaptic input. The latter may relate to changes in sensory, motor, or cognitive variables (Buzsaki, 2002; Montgomery et al., 2009) including the acquisition and storage of specific associative learning procedures, (Munera et al., 2001; Darling et al., 2011; Jurado-Parras et al., 2013). We have reported a decrease in theta coherence across the long axis of HPC that is indifferent to gross behavioral differences (e.g., running vs. REM sleep; Sabolek et al., 2009; Penley et al., 2011), but sensitive to variation in locomotor speed (Hinman et al., 2011). The present study tested the hypothesis that locomotion in novel space would alter theta across the long axis of the HPC. We compared theta and gamma indices at CA1 and DG sites in rats performing the same behavior, traveling down (1) a linear runway in a familiar space, (2) a modified path in that familiar space, and (3) a linear runway in novel space (see Figure 1A).

## Materials and methods

### Animals

Seven adult male rats (Fisher-344) were used in this study. The animals were housed on a 12-h/12-h light-dark cycle, in a temperature-controlled room. All procedures were performed in accordance with the guidelines set forth by University of Connecticut's Institutional Animal Care and Use Committee and NIH.

### Surgery

Rats were anesthetized using a ketamine cocktail (4 ml/kg; 25 mg/ml ketamine, 1.3 mg/ml xylazine, and 0.25 mg/ml acepromazine). After a midline scalp incision, small holes were drilled in the skull and four electrode arrays were positioned along the septo-temporal extent of the HPC and within the EC. Each electrode array consisted of four linearly spaced 50 μm tungsten wires such that there was a total of 16 electrodes implanted into each subject (California Fine Wire Co., Grover Beach, CA). The wires were arranged using fused silica tubing (Polymicro Tubing, Phoenix, AZ) with an outer diameter of 180 microns and implanted parallel to the coronal plane of the HPC. Three arrays were positioned at distinct septotemporal locations along the ipsilateral HPC, and a fourth array was typically positioned in EC using following coordinates (relative to bregma): AP: −2.5, −5.5, −7.5 mm; ML: 2.2, 5.5, 6; DV, 3–7.0 mm. The majority of electrodes were targeted at sites dorsal to the hippocampal fissure including positions in stratum radiatum and stratum lancunosum moleculare of CA1, as well as sites within the dorsal blade of the dentate gyrus within stratum moleculare of DG or stratum granulosum (see Figure 1D). All electrodes were attached to female pins (Omnetics, Minneapolis, MN) secured in a rectangular 5 × 4 pin array. Two skull screws positioned above the cerebellum served as indifferent and ground electrodes. The entire ensemble was secured with dental acrylic.

### Behavioral training

Prior to electrode implantation, all rats were pre-trained to alternate on an elevated linear track for food reward (chocolate sprinkles) until a criterion of at least 50 trials (with trial equal to a traversal from one end of runway to other end) within 10 min. One week post-surgery, rats were retrained to criteria on the familiar configuration maze (see Figure 1A). The maze consisted of two pivoting arms (total maze length 140 cm × 10 cm), which could be moved into 12 equally spaced positions around the center. During familiar training the maze was placed into a linear configuration. Fine grade, black, textured inserts were placed on the track and the room was maintained in bright lighting conditions. For acclimation, the rats were connected to the recording cable during all training conditions.

Following 14 days of training on the familiar configuration, electrophysiological data was collected on the 15^th^ day for the familiar condition. The next day one arm of the maze was shifted 30° and data was collected for the novel (modified path) configuration. The animals were then given one additional day of familiar maze configuration training to confirm that they maintained criteria (50 trials within a 10 min period). On the final recording day, rats were brought to a separate room (novel space). The maze was placed in a linear configuration, white, coarse grade textured inserts replaced the black inserts, and data was collected in the novel space for 1 day within a 10 min period recording session. All electrophysiological data analyses were thus restricted to recordings collected over a 10-min period on 3 separate days; one in familiar space, one on the modified path in familiar space and one in novel space.

### Data acquisition

Wide-band electrical activity was recorded (1–1894 Hz, 3787 samples/s) during each recording session using a Neuralynx data acquisition system (Bozeman, MT). Light emitting diodes attached to the headstage were tracked via a camera (33 samples/s) positioned over the track, allowing for a record of the rat's position. In order to calculate locomotor speed, the positional difference between successive tracking samples was calculated and lowpass filtered (cutoff = 0.25 Hz) in order to minimize the contribution of movement artifacts to the overall speed of the rat. A representative filtered position vs. time trace is shown in Figure 1B. Such traces were used to calculate instantaneous and mean speed during designated intervals.

### Data analysis

All data analysis was conducted using custom written programs in MatLab (The MathWorks, Natick, MA). The following criteria were applied to the acquired dataset in order to restrict all analyses to locomotor movement related data. Two criteria were set in order to identify the beginning of each trial; where a trial was defined as a single run in one direction toward the other end of the runway. First, a physical threshold 14 cm from either end of the linear track was used to eliminated data at the ends of the runway (start and ends of each trial: see Figure 1B). Second, any trial where locomotor speed decreased to below 5 cm/s was eliminated. These criteria ensured that data from intervals when the rat was stopped or turning around after consuming the food reward, or stopped or slowed during the linear traversal across the runway, were not included in the analysis. Because the length of the data was contingent on the speed of the animal (between ~ 1.5 to 2.5 s across the 140 cm runway) only the last 1.5 s of each traversal of the maze was selected for analysis. This allowed for all data segments from each trial to be the same length and 1.5 s was the fastest trial (shortest segment). This resulted in data strings of minimally 75 s (50 trials × 1.5 s) for analyses.

### Data analysis (power)

Power spectral density estimates were obtained using Welch's averaged modified periodogram method (Welch, 1967). The power for theta (6–10 Hz) and gamma (25–50 Hz and 65–100 Hz) was then calculated from the power spectral density, converted to decibels and finally normalized to 1 uV. The resulting power value and the corresponding mean speed for each non-overlapping 1.5-s segment were then subjected to a linear regression analysis.

### Data analysis (coherence)

Coherence is a measure of the linear association between two signals as a function of frequency. Coherence analysis requires longer strings of data than the power analysis. In order to calculate coherence in relation to the speed of the animal, trial data was sorted based on mean speed (from slowest trial to fastest trial). The LFP signals of the speed-sorted trials were then concatenated and then truncated into a single 20-s continuous string of data (Roark and Escabi, 1999, also see Sabolek et al., 2009; Hinman et al., 2011, 2013; Penley et al., 2011), such that each recording session (50 trials within 10 min) generated multiple 20-s long data strings with different mean speeds. Thus, the slowest trials totaling 20 s were concatenated, then next slowest totaling 20 s were concatenated and so forth for all trials, resulting in an average of 9.8 ± 0.6 separate strings of data for analysis. This procedure allows for the creation of 20 s long data strings to analyze coherence where each 20 s string is composed of segments with the same mean running speed.

Coherence values (Bullock et al., 1990) for each channel pair were computed using the Welch periodogram estimation procedure with a spectral resolution of ~2 Hz. The Welch—averaging periodogram method for estimating coherence breaks up the data into smaller blocks (~1/2 s duration), computes the coherence for each, and subsequently averages the coherence between all such blocks. To ensure that the measured coherence values were not due to chance alone, a significance estimation procedure was devised in which the coherence estimate for a pair of electrodes was compared against a null hypothesis condition consisting of signals with identical magnitude spectrum, but with zero phase coherence (Sabolek et al., 2009; Hinman et al., 2011, 2013). We generated the null hypothesis condition by randomizing the phase spectrum for each signal in the pair and subsequently computing the coherence between the two random phase signals. This procedure was bootstrapped 250 times (Efron and Tibshirani, 1993) to generate a probability distribution of frequency-dependent coherence values for the null hypothesis. This phase randomization procedure guarantees that the signal spectrums are identical but have no linear association, because the phase or time information has been removed. The coherence distribution for this null hypothesis was used to determine a threshold for each frequency band (2 Hz resolution), below which 95% of the shifted null hypothesis coherence values fell. Thus, only regions of the non-shuffled signal coherences falling above the 95% threshold were considered significant. For each channel pair, the statistically significant area in the theta (6–10 Hz), and gamma (25–50 Hz, 65–100 Hz) ranges were calculated, and normalized by the frequency range (expressed as average coherence value per Hz) to facilitate comparison of different frequency ranges. The coherence value was then normalized for bandwidth and a new zero point, the resulting normalized coherence value falls between 0 and 1.

### Statistics (linear regression analysis)

Each spectral index was separately subjected to a linear regression analysis that included the mean speed and two orthogonal dummy coded variables for each condition (modified pathway and novel space) as explanatory variables. Each electrode (power) or electrode pair (coherence) yielded a single standardized regression coefficient (β-value, where $\beta =b\frac{SDx}{SDy}$, where *b* is the slope coefficient, *SD*_x_ is the standard deviation for variable *x*, and *SD*_y_ is the standard deviation for variable *y*) for each of the explanatory variables. The resulting distributions of β-values were then individually tested for each region (DG, CA1) to determine whether that region had a mean different than zero using a *t*-test. A non-zero mean for a condition's β-value distribution indicates that the spectral index is significantly different from the familiar condition positively or negatively (controlling for any change in that spectral index over the recordings) depending on the case (Lorch and Myers, 1990). Repeated-measures ANOVA and individual *t*-tests were used to compare the distribution of β-values for each experimental condition and anatomic region.

### Histology

Following the completion of recording, rats were anesthetized with Euthasol (sodium pentobarbital solution) and transcardially perfused with ice-cold saline followed by 4% Para formaldehyde in 0.1M phosphate buffer (pH 7.2). Brains were sliced (50 μm sections) using a vibratome (Vibratome Series 1500), mounted, and Nissl stained using thionin. All electrode positions were verified and categorized according to laminar and septotemporal position (see Figures 1D,E). Septotemporal distances between electrode positions were determined by noting the location of each electrode position on a flatmap representation of the HPC (Swanson et al., 1978). Each section of a flatmap represents approximately 180 μm of tissue, and so fairly accurate approximations of the relative distance between electrodes could be determined by counting the number of sections between two electrodes. Photomicrographs of relevant electrode tracks were captured using a Nikon microscope connected to a Spot RT camera system, digitized and prepared for presentation using Adobe Photoshop 7.0.

## Results

Theta oscillations (0.2–1 mV) were confirmed both by visual inspection and power spectral density at all electrodes sites during maze running on the familiar linear track, the modified angle track (novel path in a familiar environment), and the novel linear track (linear track in a novel environment). The laminar characteristics of theta and gamma LFPs were consistent with descriptions from previous studies (Sabolek et al., 2009; Hinman et al., 2011, 2013; Penley et al., 2011).

Electrodes were positioned at sites along the septotemporal extent of CA1 (*n* = 20, where *n* = number of individual electrodes) and dentate gyrus (DG, *n* = 16). Recording sites within CA1 spanned from stratum radiatum to stratum lacunosum moleculare with the majority of sites within stratum radiatum. Following histological localization, electrode positions were plotted on a flatmap representation of the HPC (Swanson et al., 1978), with each contour line representing 180 μm (Figures 1D,E). CA1 electrode positions ranged between 1.3 and 7.6 mm from the septal pole. Within the DG, electrodes were primarily positioned in the dendritic field of the granule cells (stratum moleculare) or stratum granulosum. DG recording sites ranged between 1.4 and 5.2 mm from the septal pole.

**Figure 2:**
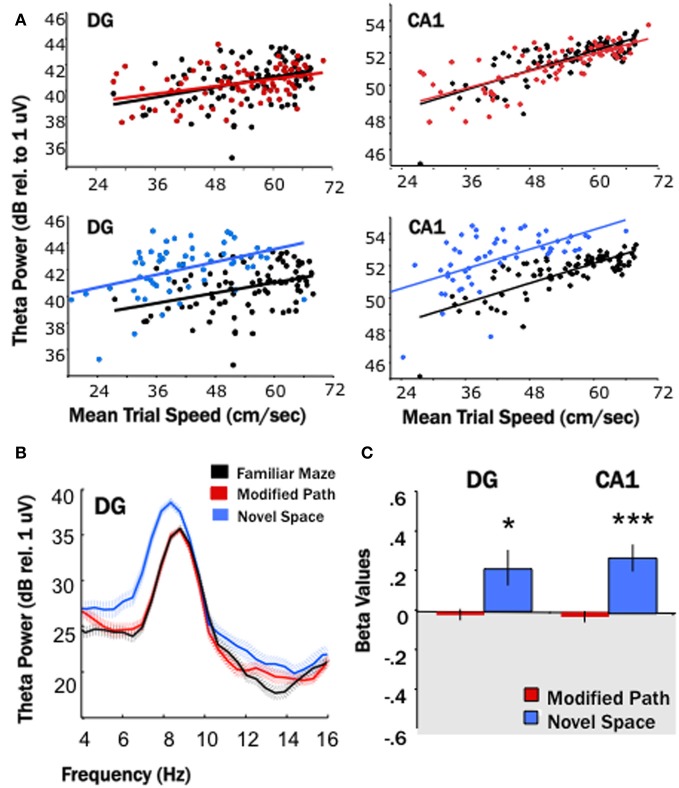
**Locomotion in a novel spatial environment increases theta power**. Theta power is higher for the novel condition (blue) than for the familiar condition (black) in both the DG and CA1. **(A)** Baseline theta power values (6–10 Hz) in CA1 and the DG as a function of the average trial speed for a single animal in the familiar condition (black), on the modified path (red), and in the novel space (blue). The distribution of speeds overlapped during the familiar and modified path conditions however the average trial speed tended to be lower while the rat locomoted through a novel space. Note the distribution of trial speeds in the novel space (blue) in both the DG and CA1 as compared to the distribution of trial speeds in the familiar condition (black). **(B)** Averaged power spectral density for a single animal across all trials during the familiar maze condition (black), on the modified path (red), and in a novel space (blue) in the DG. The hatched areas indicate the standard error of the mean across trials. Note the increase in average theta power and apparent decrease in theta frequency at the single DG site when not controlled for speed. **(C)** The standardized regression coefficient (β-value, calculated for each channel from the area of theta power and average speed of each data segment using linear regression analysis) comparing changes in the modified path (red) and novel space (blue) from the familiar condition independent of speed. There was a significant increase in theta power at DG and CA1 sites independent of changes in locomotor speed in a novel spatial environment (^*^p < 0.05, ^***^p < 0.001).

All recordings involved locomotion for food reinforcement in well-trained rats along a linear path on an open and elevated maze. All data analyses were limited to forward locomotion, eliminating the LFP signal from the ends of the runway and any period involving speed less than 5 cm/s (see Methods). Locomotor speed in novel space was characterized by slower average running speed per segment as well as longer pauses at the ends of each arm resulting in fewer total trials over the 10-min recording session, with rats running an average of 69 ± 6 trials in the familiar condition, 70 ± 7 trials on the modified path and 44 ± 9 trials in the novel space. The average speed per (1.5 s) data segment was 47.4 ± 0.5 cm/s for familiar maze and 47.9 ± 0.5 cm/s for the modified path condition. Locomotion in novel environment significantly decreased speed (44.0 ± 0.6 cm/s; *p* < 0.001; see Figures 1C, 2A) per data segment by roughly 5–10%.

Given that theta power varies as a function of locomotor speed, particularly at sites in the septal HPC (Hinman et al., 2011), the area of theta power and average speed of each trial was used in a linear regression analysis (Figure 2A) and the standardized regression coefficient (β-value) calculated for each channel. The resulting β-values were then averaged for DG and CA1 sites for both the modified path and novel space condition. The difference of the mean modified path β-values or mean novel space β-values from zero indicates whether or not there is a significant change from the familiar condition (Figure 2C).

### Effects of novelty on theta power

**Figure 3:**
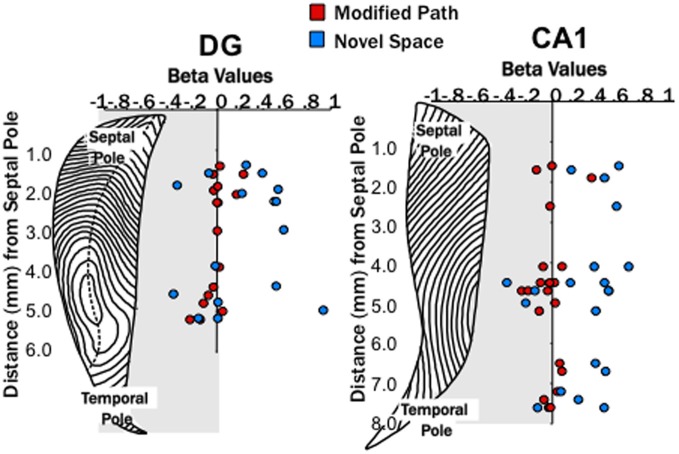
**Theta power increased at DG and CA1 sites across the entire septotemporal axis**. Distribution of β-values (calculated for each channel from the area of theta power and average speed of each data segment using linear regression analysis) from individual electrode sites within the DG (left) and CA1 (right) at different septo-temporal positions. Points indicate changes in the power of theta power (6–10 Hz) on the modified path (red) and novel space (blue) from the familiar condition when controlled for speed. Note the increases in theta power were similar at sites all along the septotemporal axis with no obvious differences in magnitude.

Our first main finding was that locomotion in a novel spatial environment was related to an increase in theta power at DG and CA1 sites (Figure 2) at all septotemporal levels of the HPC (Figure 3) independent of changes in locomotor speed. Thus, there was an increase (1–4 dBs) at the majority of sites within DG and CA1 (DG: *p* < 0.05; CA1: *p* < 0.001) when rats traversed the runway in a novel room as compared directly to the same electrode sites when the rats traversed the runway in the familiar condition (Figures 2C, 3). No significant changes were observed at these same electrode sites when the rats traversed the runway in the modified path condition (Figures 2C, 3). Thus, performing a well-learned behavior (running back and forth across a runway for food rewards) in a novel space was related to an increase in the power of the theta signal at the majority of electrode sites in the HPC.

Note that Figure 2B illustrates both the average increase in theta power and decrease in theta frequency at a single DG site, a similar change was observed at most sites when uncontrolled for speed-related alterations in power and frequency. It is important to note that theta power at septal sites is highly correlated to locomotor speed, less so at sites along the septotemporal axis (Maurer et al., 2005; Hinman et al., 2011). In contrast, the relationship of speed to frequency is fairly constant across the septotemporal axis (see Hinman et al., 2011 for references and discussion). Some, but not all of the increase in power at any electrode site can be accounted for by variation in speed. The regression analyses nonetheless indicate a significant increase in theta power independent of speed. In contrast the decrease in frequency observed at all sites could be accounted for by alterations in locomotor speed. Thus, there were no significant differences in theta frequency associated with locomotion in novel space beyond what could be attributed to variation in locomotor speed.

### Effects of novelty on theta coherence

Similar to the relationship of locomotor speed to theta power, locomotor speed is also related to theta coherence across distantly spaced electrodes (Hinman et al., 2011). The robust increase in theta power at septal sites could contribute to changes in theta coherence. Thus, as with the power analysis, a regression analysis was used to factor out the effect of locomotor speed.

**Figure 4:**
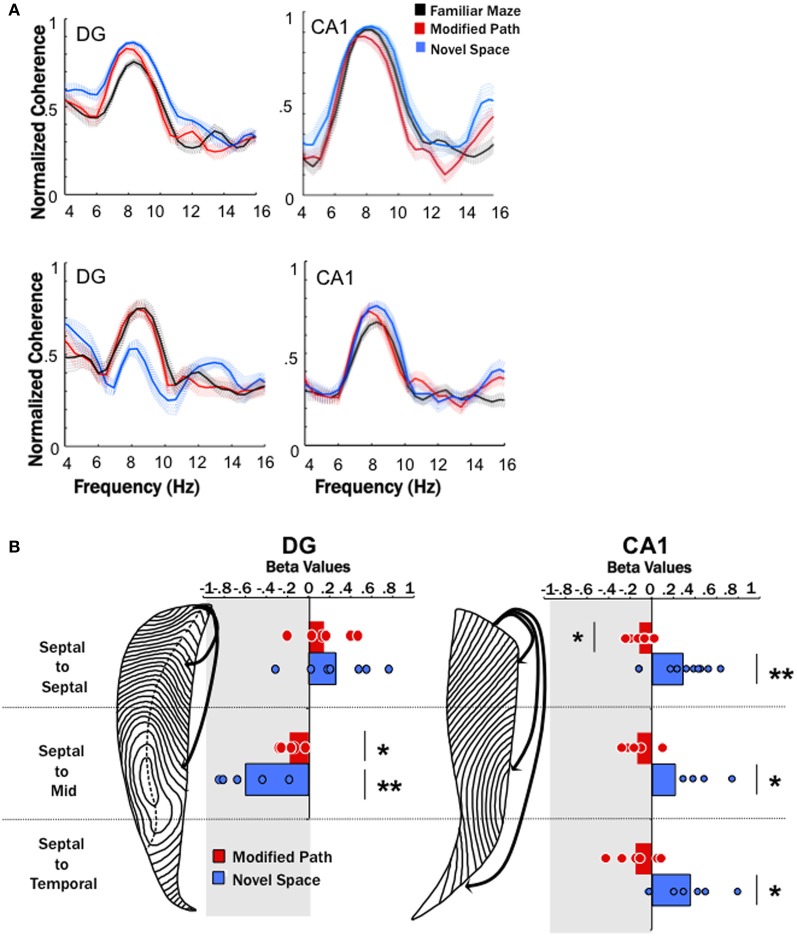
**Theta coherence decreases across the long axis of the dentate (DG) and increases across the long axis of CA1. (A)** Averaged coherence for a pair of electrodes within one animal across all data segments (trials) during the familiar maze condition (black), on the modified path (red), and in a novel space (blue) in the DG and CA1. The hatched areas indicate the standard error of the mean across trials. The top row illustrates an increase in coherence between two septal dentate sites (left) and two septal CA1 electrode sites during navigation in novel (blue) space. The bottom row illustrates a decrease in coherence between a septal and a mid-septotemporal dentate electrodes sites (left) and an increase in coherence between a septal and mid-septotemporal CA1 electrode sites (right) during navigation in novel (blue) space. Note that the raw coherence plots are not correct by speed (which is the purpose of the regression analyses). **(B)** The standardized regression coefficient (β-values) for coherence across all electrode pairs within septal, across septal, and mid-septotemporal sites and across septal and temporal sites comparing changes in the modified path (red) and novel space (blue) from the familiar condition independent of speed for DG pairs (right column) and CA1 pairs (left column). Note no significant changes were observed between mid-septotemporal-midseptotemoral pairs (data not shown) within the DG. In contrast significant increases were observed among mid-septotemporal-mid-septotemporal and temporal-temporal pairs in CA1(data not shown) (^*^p < 0.05, ^**^p < 0.01).

Our second main finding was that locomotion in a novel spatial environment was related to differential changes (DG pairs compared to CA1 pairs) in theta coherence across the septotemporal axis (Figure 4) independent of changes in locomotor speed. Briefly, theta coherence decreased between select DG electrode pairs when rats were running in novel space as compared to the familiar condition. In contrast, theta coherence increased amongst most CA1 electrode pairs during running in novel space as compared to the familiar condition.

No differences were observed among nearby dentate electrode pairs (e.g., septal to septal or mid-septoemporal to mid-septotemporal) in either the modified or novel space condition when compared to the familiar condition. In contrast, we observed a prominent decrease in coherence (range 0.1–0.5) between septal and mid-septotemporal electrode pairs (2–5 mm distant along the septotemporal axis (*p* < 0.01; Figure 4B) in the DG during running in novel space.

In contrast to the changes observed at dentate pairs, we observed significant increases in theta coherence (range 0.1–0.2) within the CA1 region between most electrode pairs during locomotion in the novel condition (*p* < 0.001; Figure 4B) as compared to the familiar condition. The increase in coherence was fairly consistent across CA1 electrode pairs that spanned different distances across septotemporal space (septal to septal: β = 0.29 ± 0.08; septal to mid septal: β = 0.47 ± 0.10; septal to temporal: β = 0.35 ± 0.12; Figure 4B). As might be expected the largest increases in coherence were observed among pairs with relatively low coherence values in the familiar condition (e.g., 0.5–0.6), while electrode pairs with larger coherence values (>0.7) exhibited more modest increases.

It should also be noted that we observed a moderate decrease at select DG electrode pairs (septal to mid septal: *p* < 0.05; Figure 4B) during performance of the modified path task as compared to the familiar task. Similarly, theta coherence significantly decreased, though by a lesser amount than in the novel environment, between septal CA1 electrode pairs (*p* < 0.01; Figure 4B) when running on the modified path as compared to the familiar task. Further there was a fairly consistent trend for decreased coherence amongst most CA1 pairs (see Figure 4B). Thus, some aspects of the theta signal may be sensitive to changes in directionality (the modified path condition involved a roughly 30° left turn). Such findings require further elucidation particularly if differential turns are used as indices of cognitive performance (e.g., T-maze performance).

### Effects of novelty on gamma power

**Figure 5:**
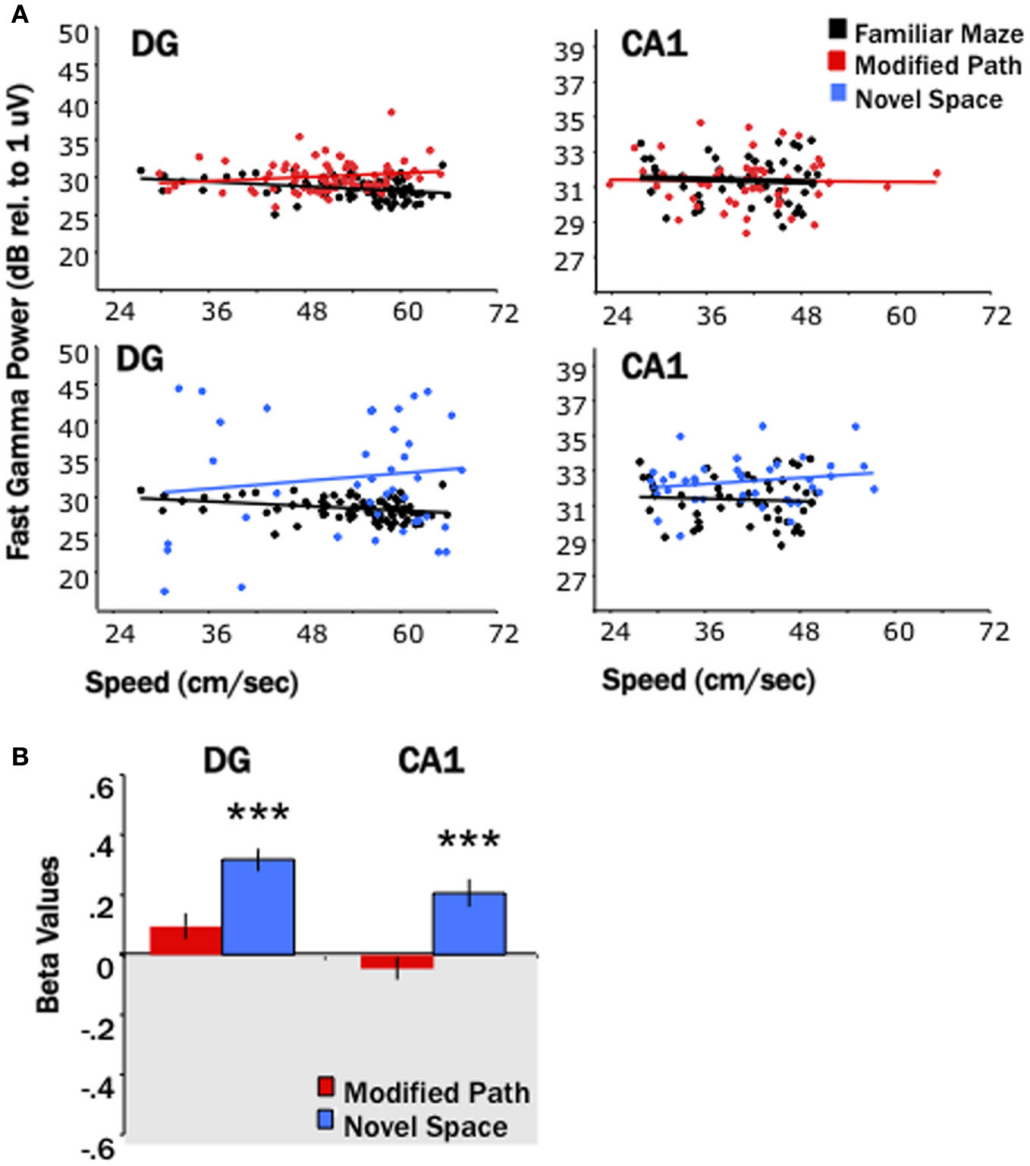
**Locomotion in a novel space increases fast gamma power. (A)** Baseline fast gamma power values (integrated across 65–100 Hz) in CA1 and the DG as a function of the locomotor speed for a single animal in the familiar condition (black), the modified path condition (red), and in the novel space (blue). Power is presented in dB relative to 1 uV. **(B)** The standardized regression coefficient (β-values) for fast gamma power comparing changes in the modified path (red) and novel space (blue) from the familiar condition independent of speed (^***^p < 0.001).

**Figure 6:**
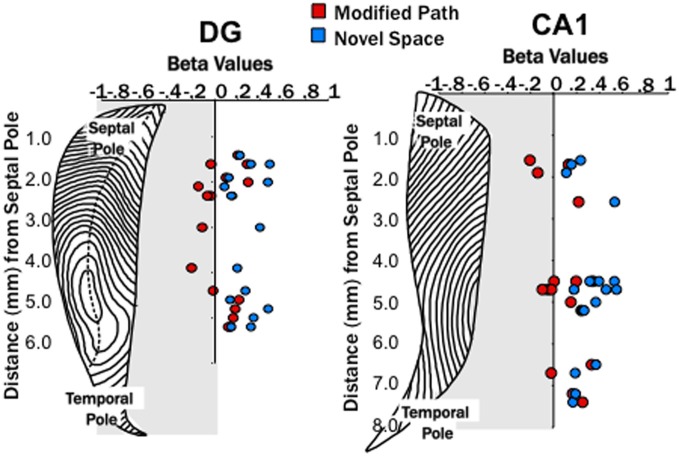
**Locomotion in a novel spaces increases fast gamma power across both the dentate and CA1**. Distribution of β-values for fast gamma power and average speed of each trial (using linear regression analysis) across the septo-temporal axis of the DG and CA1. Points indicate changes in the power of fast gamma power (65–100 Hz) on the modified path (red) and novel space (blue) from the familiar condition when controlled for speed. Changes in gamma power at DG and CA1 sites did not differ significantly along the septo-temporal axis.

Similar to increases in theta power in relation to locomotor speed, Chen et al. (2011) have recently reported increases in gamma in relation to speed. Researchers have focused on two segregate gamma regimes (slow 25–50 Hz, fast 65–100 Hz) that may reflect differences in the mechanisms and/or synaptic inputs by which they are generated (Colgin et al., 2009). Our third main finding was that locomotion in a novel spatial environment was related to increases in “fast” gamma power at most DG and CA1 sites (Figures 5, 6) independent of changes in locomotor speed. Thus, fast gamma power significantly increased during locomotion under the novel space condition at electrode sites within CA1 and the DG (*p*'s < 0.001; Figure 5). The increase in gamma power was similar at sites within the dentate and CA1 at all septotemporal levels of the HPC (CA1 septal: β = 0.16 ± 0.08; mid-septotemporal: β = 0.25 ± 0.06; temporal β = 0.24 ± 0.04; DG septal: β = 0.29 ± 0.04; mid-septotemporal: β = 0.37 ± 0.07; Figure 6). No significant changes in gamma power were observed during the modified path condition (Figures 5, 6). In contrast to the increases observed in “fast” gamma, there were no changes in slow gamma power on either the novel space or modified path conditions for either CA1 or the DG (data not shown).

### Effects of novelty on gamma coherence

In association with an increase in gamma power in CA1, our fourth main finding was that locomotion in a novel spatial environment was related to increased coherence across the CA1 axis in the “fast” gamma regime (Table 1) independent of changes in locomotor speed.

**Table 1:** **Gamma coherence by septotemporal region**.

	**Fast gamma band**		**Slow gamma band**	
	**Dentate gyrus**	**CA1**	**Dentate gyrus**	**CA1**
Entire septo-temporal axis	(*n* = 12)	(*n* = 20)	(*n* = 12)	(*n* = 20)
Modified path	0.05 ± 0.09	0.01 ± 0.05	0.07 ± 0.09	−0.10 ± 0.04
Novel space	0.05 ± 0.09	0.35 ± 0.08^**^	0.02 ± 0.11	−0.10 ± 0.07
Septal	(*n* = 7)	(*n* = 10)	(*n* = 7)	(*n* = 10)
Modified path	0.19 ± 0.13	−0.06 ± 0.07	0.21 ± 0.11	−0.13 ± 0.07
Novel space	0.12 ± 0.14	0.21 ± 0.10	0.21 ± 0.12	−0.23 ± 0.07
Mid septotemporal	(*n* = 5)	(*n* = 4)	(*n* = 5)	(*n* = 4)
Modified path	−0.14 ± 0.05	−0.04 ± 0.05	−0.13 ± 0.11	−0.11 ± 0.08
Novel space	−0.03 ± 0.09	0.55 ± 0.12^*^	−0.23 ± 0.14	−0.06 ± 0.11
Temporal		(*n* = 6)		(*n* = 6)
Modified path		0.15 ± 0.08		−0.03 ± 0.06
Novel space		0.44 ± 0.17^*^		0.09 ± 0.14

*All values expressed as beta values compared to the familiar maze condition. Fast gamma band includes *65–100* Hz. Slow gamma band includes 25–50 Hz*.

*, ** *p < *0.05, 0.001*, respectively. n = number of electrode pairs*.

Despite the increase in gamma power, no increase in gamma coherence was observed among dentate sites positioned across the septotemporal axis during navigation in novel space. In contrast, there was an increase in fast gamma coherence across distant CA1 electrodes that was most prominent between more distant electrode pairs (septal to septal: β = 0.21 ± 0.10: septal to mid septal: β = 0.55 ± 0.12; septal to temporal: β = 0.44 ± 0.17). There were also no differences in slow gamma coherence between the familiar and novel or modified path conditions (Table 1).

## Discussion

Despite obvious septotemporal differences, the variables that systematically engage the HPC across the long axis or segregate the physiological activity of hippocampal circuitry are not well defined. In this regard, one might expect that novel spatial experiences might selectively engage the septal pole of the HPC and temporally isolate neurophysiologic activity across the long axis of the HPC. Alternately, one might expect that novel spatial information synchronize theta across the long axis, engaging the entirety of the HPC in processing any experience. Analysis of theta signals within the HPC and across structures (e.g., Jones and Wilson, 2005), similar to analysis of variations in the blood-oxygen-dependent (BOLD) signal used in functional neuroimaging (Law et al., 2005; see also Logothetis and Wandell, 2004 for review), could reveal the engagement of distributed neural circuits in common processing tasks.

The present study tested the hypothesis that locomotion in novel space would alter theta and/or gamma signals across the septotemporal (long) axis of the HPC. Our findings demonstrate alterations in the power and coherence of both theta and “fast” gamma signals across the long axis. Kocsis et al. (2007) have reported increases in theta power in relation to exploratory behavior without specifically examining locomotor speed. Our findings reveal that rats performing a well-learned behavior, navigating across a runway in a novel space, exhibited alterations in theta power and coherence independent of any alterations of locomotor speed. As importantly, we report increases in theta and “fast” gamma power at sites across the septotemporal axis, suggesting a more uniform engagement of hippocampal circuits across the long axis during the processing of a novel spatial experience. Our findings suggest that certain conditions promote functional integration of hippocampal activity despite well-recognized septotemporal differences in the underlying anatomy.

### LFP power and coherence indices

Our interest is largely in assessing the utility of theta and gamma LFP signals as indices of function and defining the conditions that may integrate (synchronize) or segregate the activation of HPC circuits across the long axis. The primary source of power and coherence changes in LFP signals is alterations in synaptic input. In this regard, we suggest that increased power at individual sites and increase coherence between sites, reflect an increase in temporally specific synaptic input impinging on the somatodendritic field of HPC neurons. Increases in power likely reflect increased synchrony in the subset of inputs impinging at a particular location, while increases in coherence likely reflect increased synchrony in the broader (independent or overlapping) subsets impinging on both sites. While LFP signals are not solely defined by synaptic input (e.g., EPSPs, IPSPs), as alterations in intrinsic cellular properties (e.g., oscillatory membrane potentials) certainly contribute (see Buzsaki, 2002; Logothetis and Wandell, 2004 for a broad review), synchronized intrinsic oscillations in large number of neurons would need to be engaged by synaptic input.

The “novelty” related inputs needed to bring about large-scale increases in power and/or coherence need not be directly attributed to increase synchrony in the primary excitatory (e.g., entorhinal or CA3/mossy cell inputs) or inhibitory (e.g., GABAergic basket cells) input. Rather they could reflect an increase in neuromodulatory input(s). Thus, for example, increased acetylcholine release could enhance the post-synaptic response to either excitatory or inhibitory input (see Cole and Nicoll, 1983; Hasselmo, 2006) and could drive the rhythmic discharge of local GABAergic basket cells (see Nagode et al., 2011). We suggest, that, within limits, greater power, and greater coherence reflect a numerically larger, and temporally more precise, network engaged in a common process; in this case, the CA1 network engaged in encoding the features of a novel spatial environment and increasing subsequent retention (see Nyhus and Curran, 2010 for review).

In particular, the theta signal increased in amplitude at the CA1 electrodes. The increase in amplitude was concomitant to an increase in theta synchrony (coherence) across septotemporally distant CA1 electrode pairs. The increase in coherence is not necessarily related to an increase in power, although synchronous increases in power likely contribute to increased coherence. The present study also revealed that the theta signal increased in power across the DG electrodes, while theta coherence actually decreased across distant DG electrode pairs. The contrasting organization of entorhinal inputs to the dendritic field of CA1 neurons as compared to the dentate (see Amaral and Witter, 1989) may contribute to this difference. Briefly, medial (MEA) and lateral entorhinal (LEA) inputs are organized laminarly with every DG neuron receiving inputs from both MEA and LEA neurons; in contrast, MEA and LEA inputs are distributed areally in the much broader proximodistal axis of CA1 (CA3 border being proximal and subicular border being distal). Henriksen et al. (2010) have reported differences in the spatial tuning of CA1 neurons across the proximodistal axis and we are currently examining proximodistal differences in theta LFPs across CA1, particularly with regards to how locomotor speed influences changes in theta power. The increase in theta and gamma power at DG electrodes with a corresponding and substantive decrease in theta coherence along the long axis requires further analysis. Nonetheless, our findings indicate that novel sensory (spatial) information synchronizes the theta and gamma-related afferent activation of hippocampal neurons along the septotemporal extent of the CA1 network of neurons and this suggests synchronization, rather than segregation, of functional activity across the CA1 axis in relation to novelty.

### Theta vs. “fast” (65–100Hz) gamma

Rhythmic LFP signals such as theta and gamma are thought to serve as clocking mechanisms for bringing distributed ensembles together in time, as well as temporally isolating or differentiating cell assemblies (Llinas et al., 1991, 2005; Gray, 1994; Buzsaki and Chrobak, 1995; Csicsvari et al., 2003). The recorded LFP signals however, actually reflect synchronized membrane events brought about by a vast ensemble of synaptic inputs. In this respect the “clocking mechanism” is built into the neurons and neuronal circuits rather than provided by a “clock” nucleus (e.g., suprachiasmatic nucleus). Theta LFP signals, in CA1, largely reflect the interactions of entorhinal and CA3 synaptic inputs within the dendritic field of CA1 neurons as sculpted by somatodendritic inhibition from theta coupled higher frequency gamma inputs such as fast-firing GABAergic basket cells (see Buzsaki et al., 1983; Bragin et al., 1995; Chrobak and Buzsaki, 1998; Belluscio et al., 2012). The latter, fast-frequency GABAergic input, are not the sole source of rhythmic gamma potentials. Gamma frequency signals likely derive from multiple interacting inputs that synchronize/suppress and isolate distinct compartments of CA1 neurons (see Belluscio et al., 2012 for recent discussion of the source and frequency segregation of “gamma”).

The rhythmic excitatory (entorhinal and CA3) inputs at theta frequency serve as the predominant excitatory drive to the dendritic field of CA1 pyramidal neurons (contributing to the theta LFP signal). These excitatory inputs concurrently drive a diverse network of GABAergic neurons including the GABAergic basket cell networks that regulate the somatic and dendritic compartments of CA1 neurons. Colgin et al. (2009) have emphasized the distinction between “slow” and “fast” frequency gamma, the phase-relationship of each to distinct portions of the theta cycle and their respective coherence to gamma signals in the CA3 (slow) and MEA (fast). The distinctive phase-relationship of each as well as the frequency differentiation, likely supports the temporal arrival of CA3 inputs in advance of entorhinal inputs (see Ang et al., 2005 or Colgin et al., 2009 for discussion) as well as the accelerating excitatory drive on GABAergic neurons within each theta “wave” of excitatory input (driving the basket cells to discharge at faster frequencies). We examined both slow and fast gamma and observed a uniform increase in “fast” gamma power at CA1 and DG sites and increased “fast” gamma coherence across distant CA1 pairs along the long axis without any changes in “slow” gamma.

## Summary

Increases in the theta in particular has been noted in relation to a number of sensory, motor, and cognitive variables during task performance as well as during the acquisition of associative learning tasks (e.g., Munera et al., 2001; Buzsaki, 2002; Darling et al., 2011; Jurado-Parras et al., 2013) and have been linked to successful encoding and subsequent retention of memories (see Nyhus and Curran, 2010 for review). Several studies have indicated significant changes in theta and gamma coherence across laminar-specific sites in the septal HPC as a function of state and information processing (Jones and Wilson, 2005; Kay, 2005; Martin et al., 2007; Montgomery et al., 2008, 2009). Similarly, a number of human and animal studies have demonstrated a relationship between theta power and coherence, as well as gamma and theta/gamma coupling at both medial temporal lobe and other neocortical sites in relation to information processing (Fell et al., 2001, 2003; Sederberg et al., 2003; Canolty et al., 2006; Tort et al., 2009; Shirvalkar et al., 2010). In the present study, the same rats performed the same behavioral task (running on a linear runway) with no distinct cognitive demands save the experience of encoding novel sensory (spatial) information. We report increases in the power and coherence of theta and “fast” gamma across the septotemporal extent of the CA1 network. These findings suggest that environmental novelty synchronizes and engages the entirety of the septotemporal axis to encode novel sensory (spatial) experience.

### Conflict of interest statement

The authors declare that the research was conducted in the absence of any commercial or financial relationships that could be construed as a potential conflict of interest.
